# Supplementary material for: Kinetic Modelling of [68Ga]Ga-DOTA-Siglec-9 in Porcine Osteomyelitis and Soft Tissue Infections
Source: Molecules. 2019 Nov 13;24(22):4094. doi: 10.3390/molecules24224094 (PMC6891593; doi:10.3390/molecules24224094)
Supplement: Supplementary file 1 [file molecules-24-04094-s001.pdf]

Supplemental material for:

Kinetic modelling of [^{68}Ga]Ga-DOTA-Siglec-9 in porcine osteomyelitis and soft tissue infections

by Lars Jødal et al.

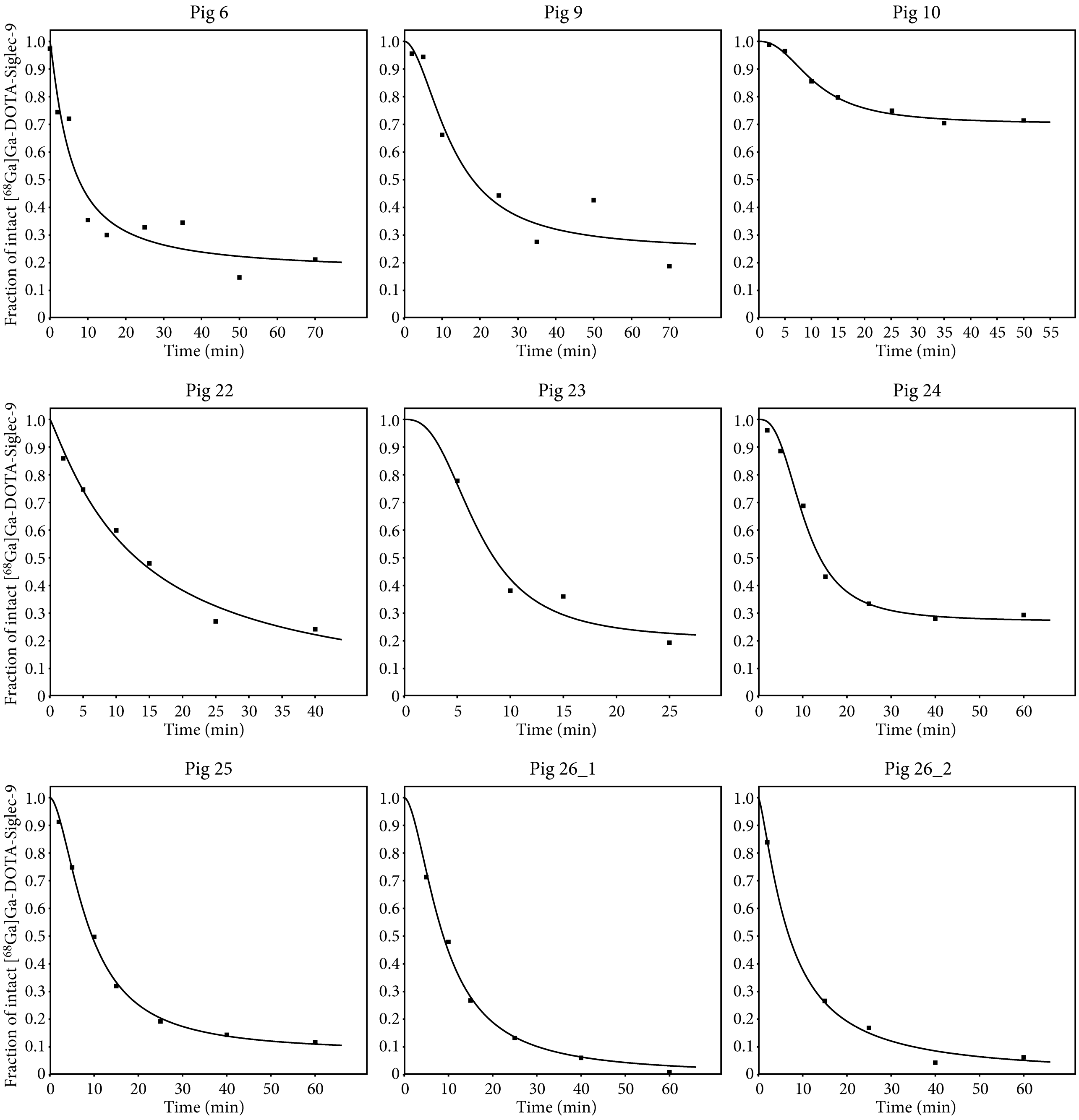

Figure S1. Data points and curve fits for the fraction of intact [^{68}Ga]Ga-DOTA-Siglec-9 (parent tracer fraction).

Note: It is unexplained why metabolism was much less pronounced in pig 10 than in the other pigs.

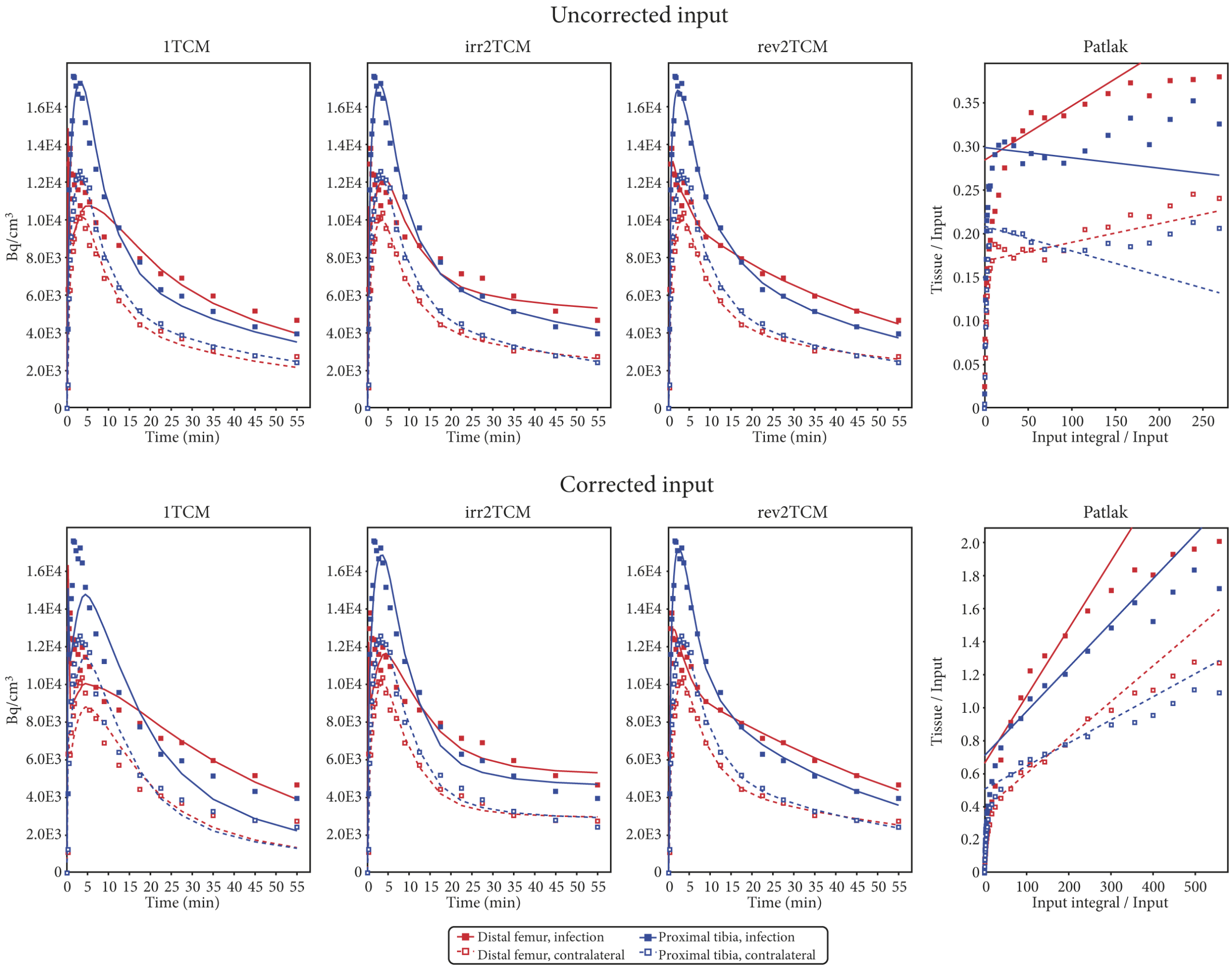

Figure S2. Model fits and Patlak plots for pig no. 6. For details of the modelling procedure, see the Methods section of the paper. Pig no. 6 had bone infections in the distal femur (red) and the proximal tibia (blue).

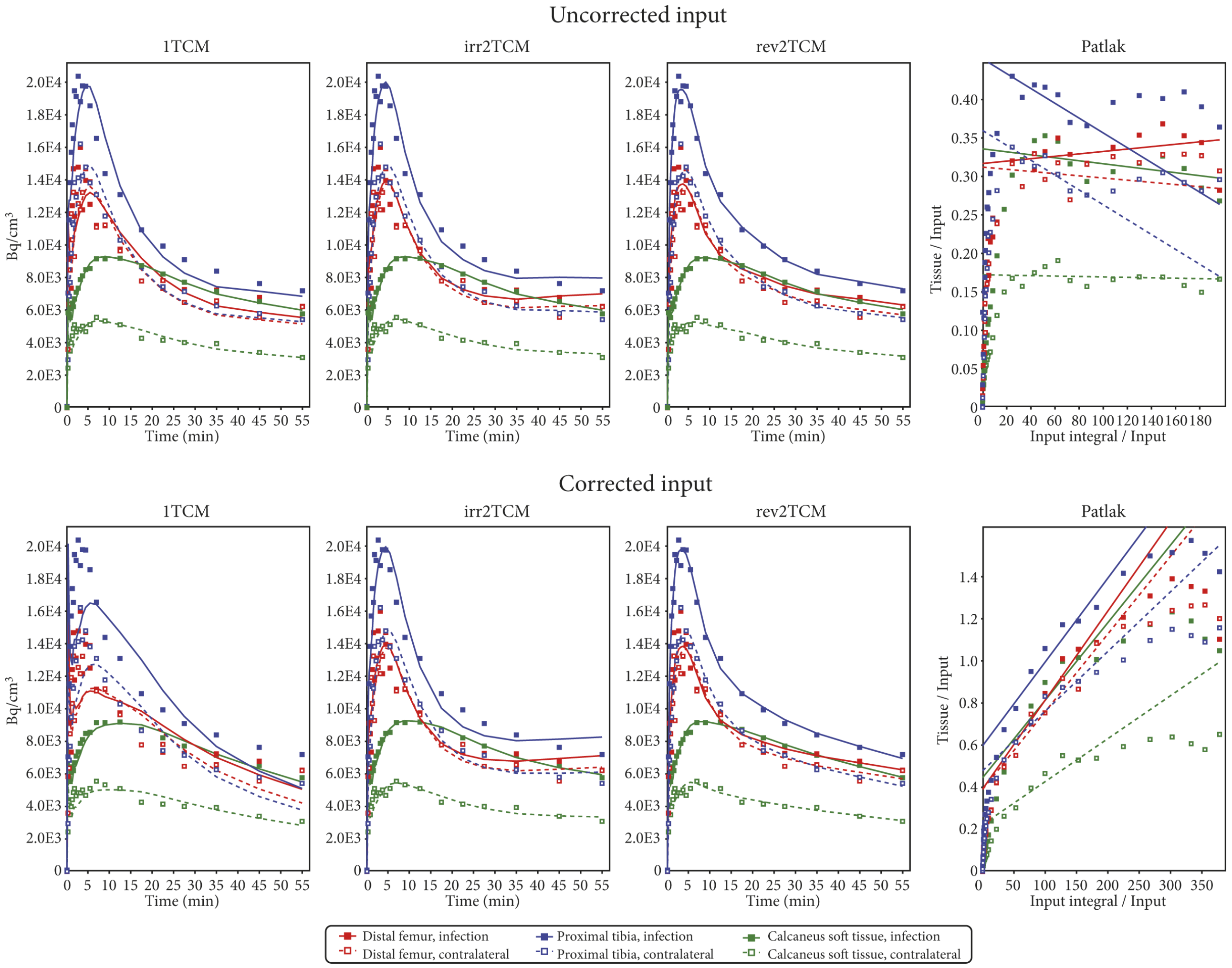

Figure S3. Model fits and Patlak plots for pig no. 9. Pig no. 9 had bone infections in the distal femur (red) and proximal tibia (blue) and a soft tissue infection related to the calcaneus (green).

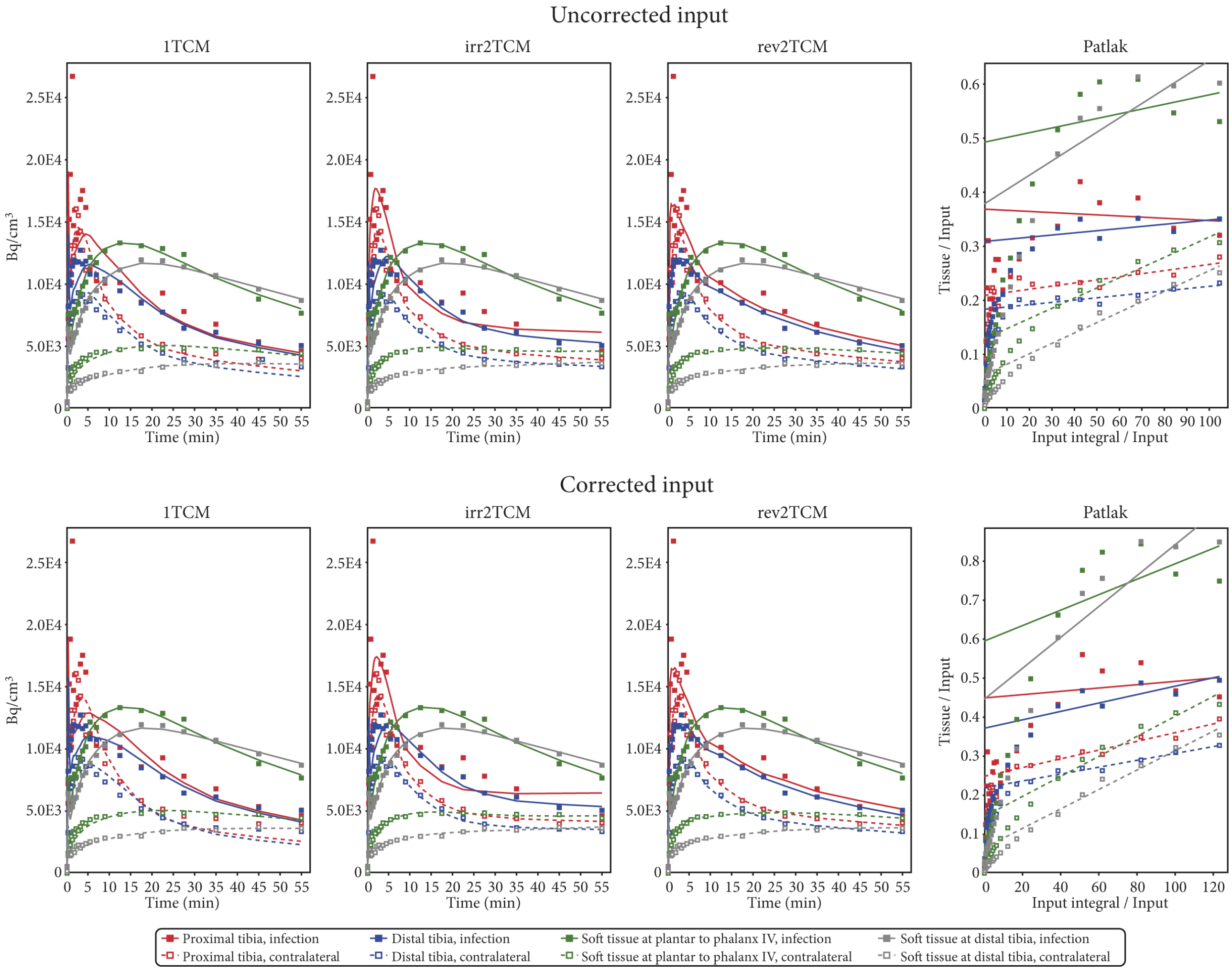

Figure S4. Model fits and Patlak plots for pig no. 10. Pig no. 10 had bone infections in the proximal (red) and distal tibia (blue); soft tissue infections included an abscess in the foot tissue plantar to phalanx IV (green) and soft tissue at the distal tibia (grey).

Note: In pig no. 10, the data indicated only little metabolism of the tracer (cf. Figure S1), and therefore the difference between the uncorrected and the corrected input curve was relatively small for this animal.

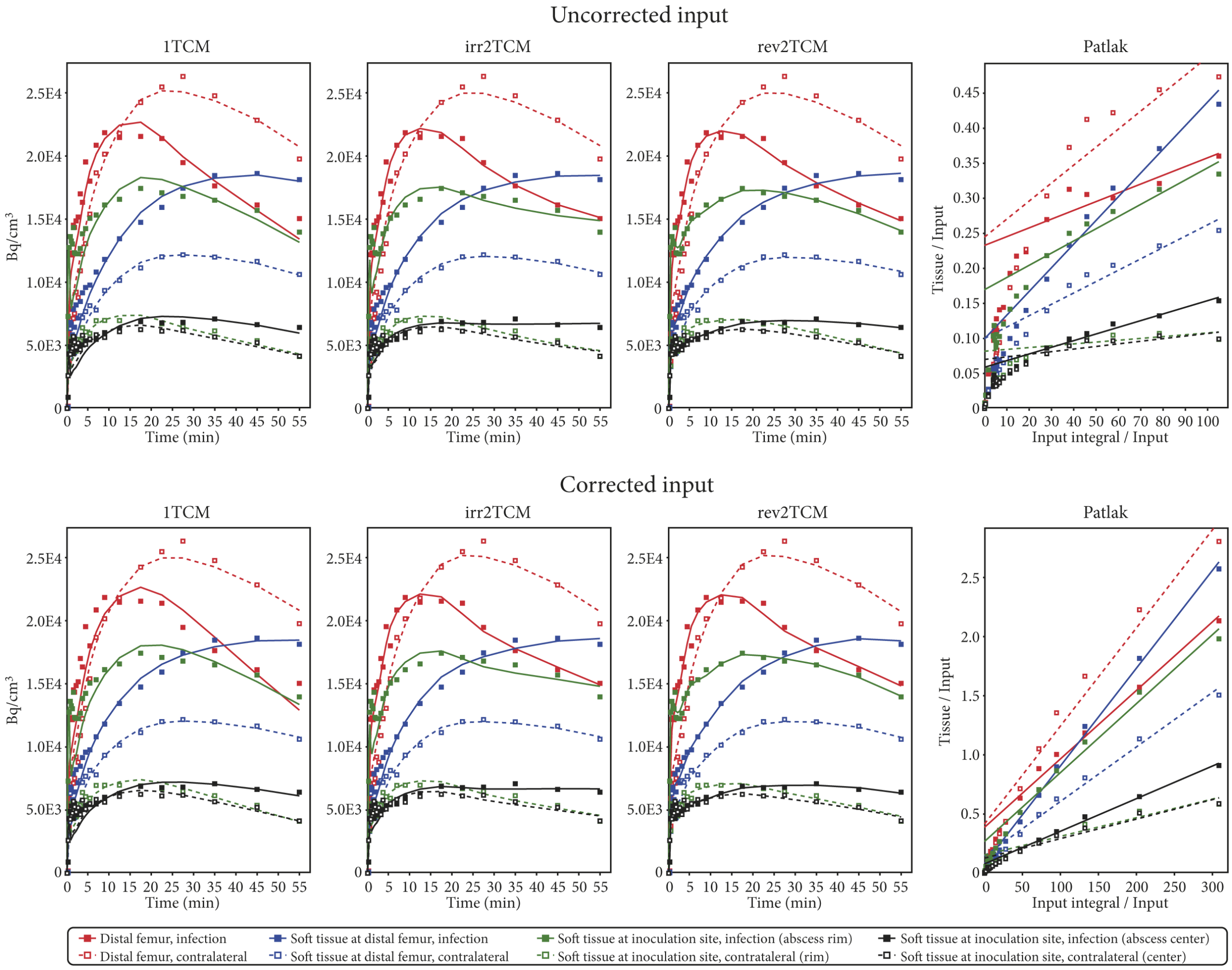

Figure S5. Model fits and Patlak plots for pig no. 22. Pig no. 22 had a bone infection in the distal femur (red curve). Soft tissue infections included tissue at the distal femur (blue curve) and an abscess at the inoculation site; for the abscess, VOIs were drawn at both the rim (green curve) and the “cold” centre (black curve).

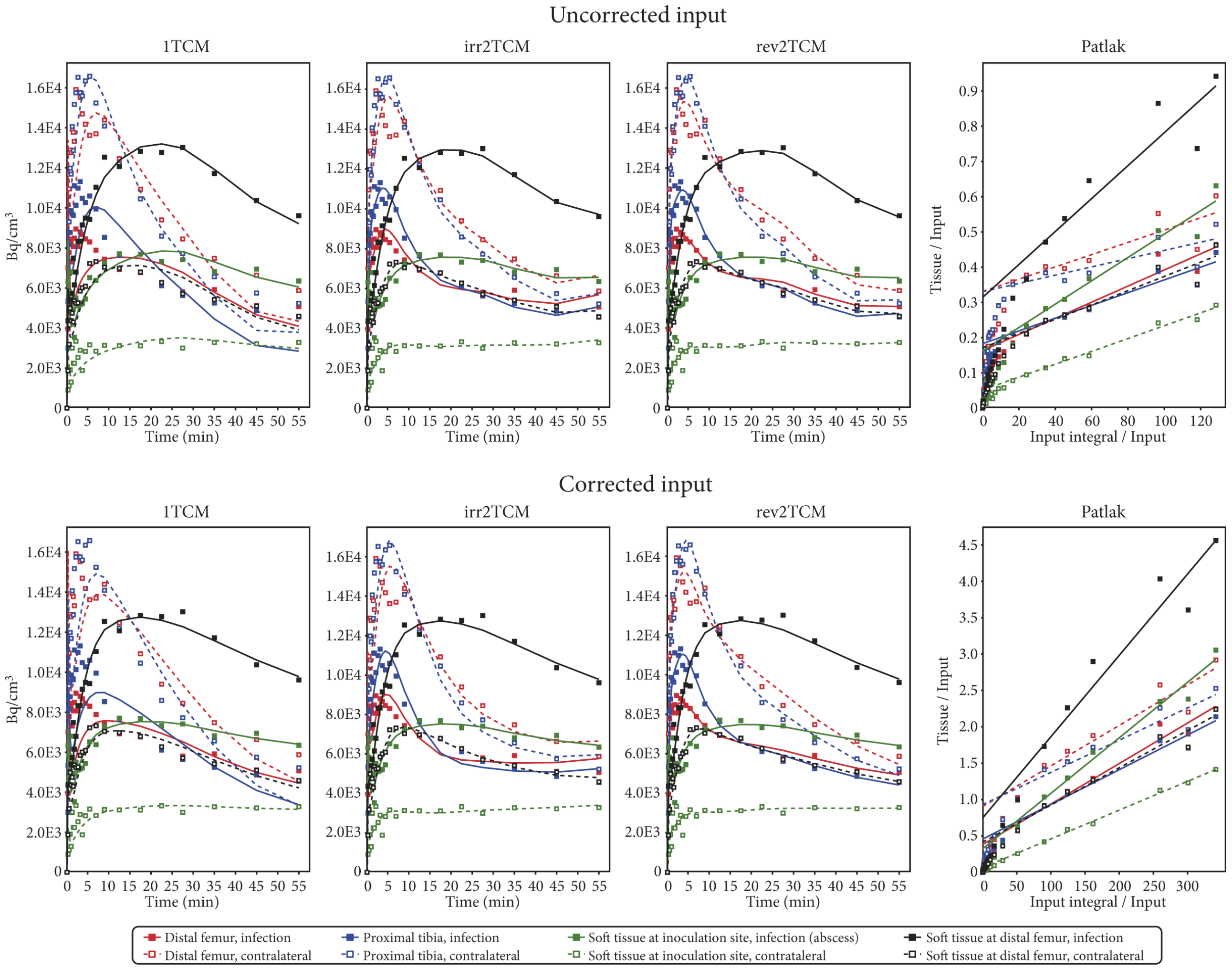

Figure S6. Model fits and Patlak plots for pig no. 23. Pig no. 23 had bone infections in the distal femur (red curve) and the proximal tibia (blue curve), and soft tissue infections at the inoculation site (green curve) and at the distal femur (black curve).

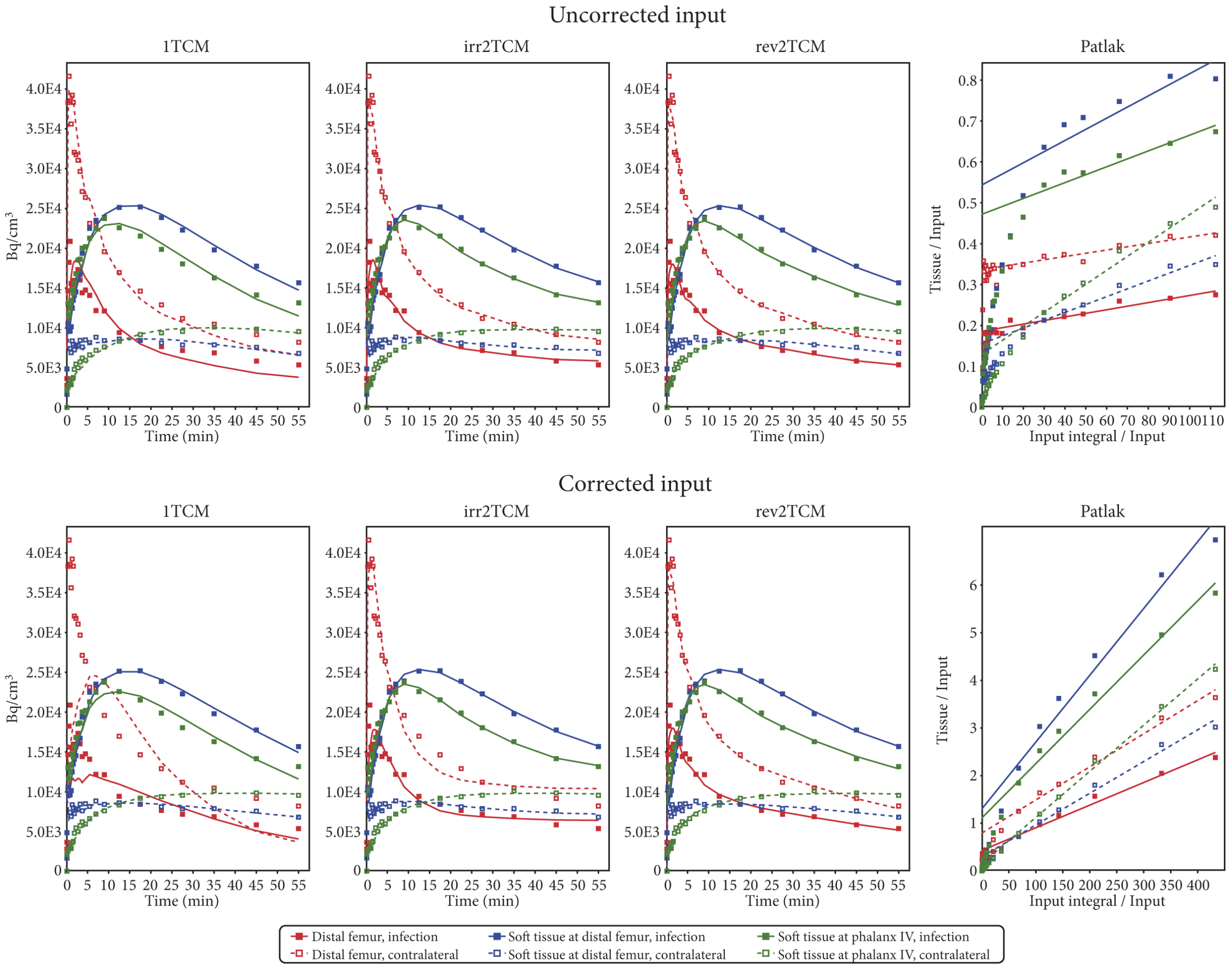

Figure S7. Model fits and Patlak plots for pig no. 25. Pig no. 25 had a bone infection in the distal femur (red), and soft tissue infections at the distal femur (blue) and in the foot at phalanx IV (green).

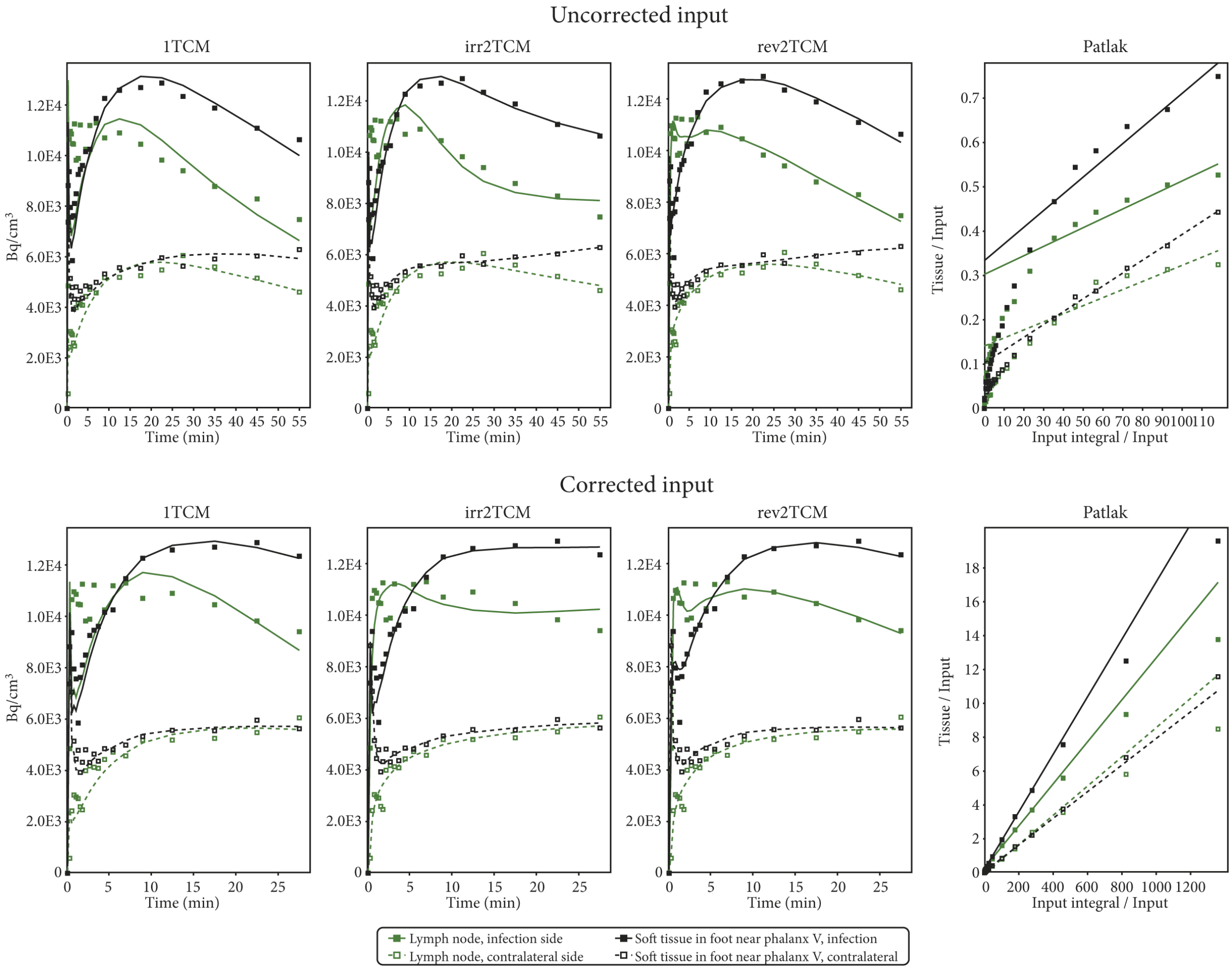

Figure S8. Model fits and Patlak plots for pig no. 26 (first scan). As noted in the Methods section of the paper, modelling with the corrected input function was based only on the data from 0 to 30 minutes for this pig. Pig no. 26 had soft tissue infection in a superficial popliteus lymph node (green) and in the foot in tissue near phalanx V (black).

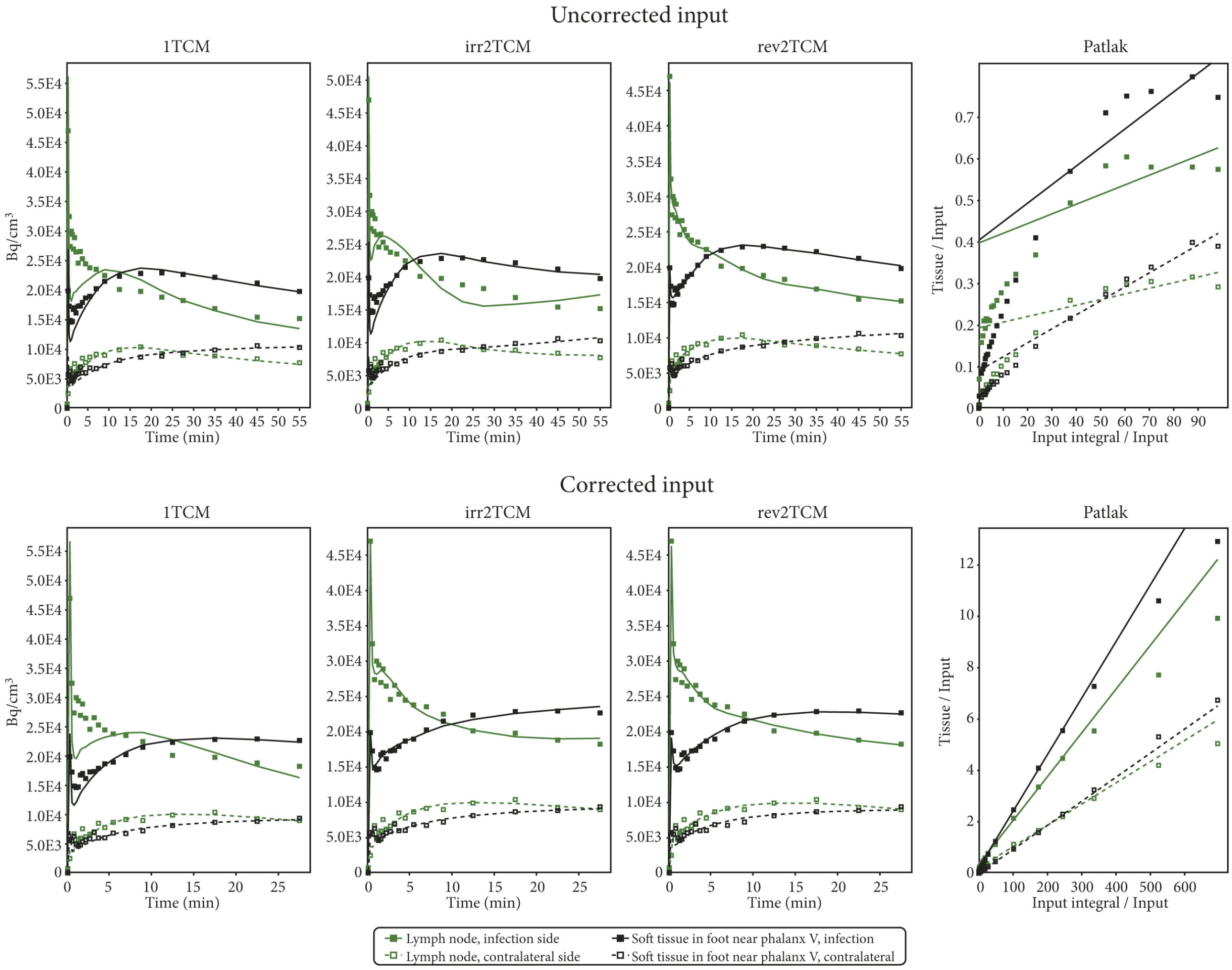

Figure S9. Model fits and Patlak plots for pig no. 26 (second scan). This scan was performed after the injection of "cold" Siglec-9; see the Methods section of the paper. VOIs in this scan were drawn in the same positions as in Figure S8 (first scan of the same pig).

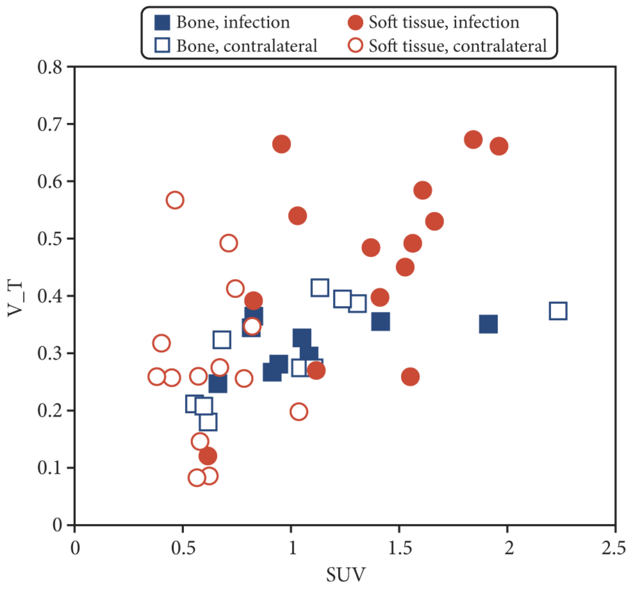

Figure S10. Volume of distribution (V_T) as a function of standardized uptake value (SUV).

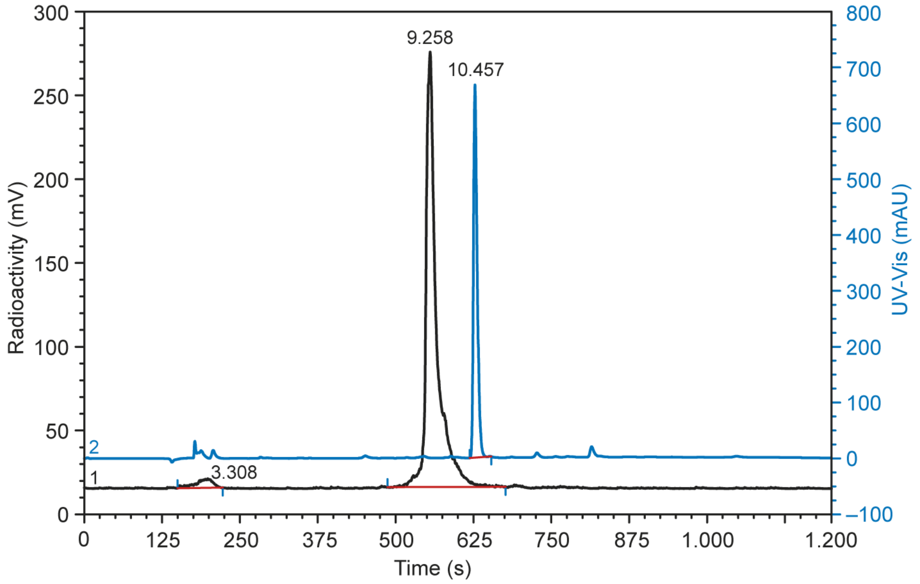

Figure S11. Radio-HPLC and UV analysis from [^{68}Ga]Ga-DOTA-Siglec-9 production. The horizontal axis shows time in seconds, vertical axis shows signal strength. The black curve is the radioactivity signal with the [^{68}Ga]Ga-Siglec-9 signal peaking at 9.258 min (555 s) and a small impurity signal peaking at 3.308 min (198 s). To get a UV-Vis signal, the radiotracer sample was spiked with unlabelled (and therefore non-radioactive) DOTA-Siglec-9 in much higher quantity than [^{68}Ga]Ga-DOTA-Siglec-9. The blue curve shows the UV signal, with the dominating signal from DOTA-Siglec-9 peaking at 10.457 min (627 s). As the unlabelled DOTA-Siglec-9 is more polar than the gallium-complexed tracer, the unlabelled molecule has longer retention time than the radiotracer.

Table S1. Volumes of the VOIs used in kinetic modelling

Pig no.	Lesion	VOI volumes in cm^3	
		Infection VOIs (right limb)	*Control VOIs (left limb)*
6	Distal femur	6.0	5.2
9	Distal femur	2.9	2.0
22	Distal femur	1.3	1.2
23	Distal femur	2.9	2.5
25	Distal femur	0.6	0.7
6	Proximal tibia	1.0	3.2
9	Proximal tibia	2.2	2.2
10	Proximal tibia	1.4	2.8
23	Proximal tibia	2.3	2.2
10	Distal tibia	2.3	2.4
22	Soft tissue at distal femur	2.2	2.5
23	Soft tissue at distal femur	2.0	2.8
25	Soft tissue at distal femur	1.5	1.5
10	Soft tissue at distal tibia	5.7	2.8
9	Abscess at calcaneus	4.5	2.0
10	Abscess plantar to phalanx IV	3.7	2.8
25	Abscess plantar to phalanx IV	0.7	0.7
26	Soft tissue plantar to phalanx V	1.1	0.9
26 *	Soft tissue plantar to phalanx V	1.0	1.0
22	Abscess at inoculation	3.8	4.1
23	Abscess at inoculation	2.0	2.5
22	Centre of abscess at inoculation	2.9	2.8
26	Lymph node	1.1	1.1
26 *	Lymph node	1.1	0.9

* Second scan of pig no. 26 (see main text)

Table S2. Comparison of models 1TCM/irr2TCM/rev2TCM, using the *uncorrected* input functions. For each VOI, the difference from the minimum value, $\Delta AIC_c = AIC_c - AIC_{c,min,}$, is tabulated. For a given data set, the model with $\Delta AIC_c = 0$ (i.e., the lowest AIC_c value) is theoretically preferable.

Pig no.	Lesion	*Infection VOIs (right limb)*			*Control VOIs (left limb)*		
		**1TCM**	**irr2TCM**	**rev2TCM**	**1TCM**	**irr2TCM**	**rev2TCM**
6	Distal femur	61	51	0	23	0	3
9	Distal femur	25	11	0	17	5	0
22	Distal femur	21	0	3	0	3	6
23	Distal femur	68	28	0	36	8	0
25	Distal femur	36	3	0	24	0	2
6	Proximal tibia	38	27	0	0	3	6
9	Proximal tibia	52	43	0	29	24	0
10	Proximal tibia	16	9	0	17	0	3
23	Proximal tibia	76	10	0	49	3	0
10	Distal tibia	45	34	0	37	0	1
22	Soft tissue at distal femur	26	24	0	10	11	0
23	Soft tissue at distal femur	15	4	0	41	2	0
25	Soft tissue at distal femur	20	0	3	32	29	0
10	Soft tissue at distal tibia	0	3	5	18	13	0
9	Abscess at calcaneus	5	8	0	10	10	0
10	Abscess plantar to phalanx IV	0	3	6	53	35	0
25	Abscess plantar to phalanx IV	22	0	2	33	28	0
26	Soft tissue plantar to phalanx V	20	6	0	15	0	3
26 *	Soft tissue plantar to phalanx V	49	50	0	28	27	0
22	Abscess at inoculation	74	58	0	16	16	0
23	Abscess at inoculation	10	0	3	25	1	0
22	Centre of abscess at inoculation	58	42	0	27	26	0
26	Lymph node	53	41	0	15	17	0
26 *	Lymph node	56	46	0	22	17	0

* Second scan of pig no. 26 (see main text)

Table S3. As in Table S2 but with the *corrected* input functions.

Pig no.	Lesion	*Infection VOIs (right limb)*			*Control VOIs (left limb)*		
		**1TCM**	**irr2TCM**	**rev2TCM**	**1TCM**	**irr2TCM**	**rev2TCM**
6	Distal femur	53	44	0	69	17	0
9	Distal femur	41	12	0	40	6	0
22	Distal femur	40	0	2	0	3	6
23	Distal femur	67	45	0	41	22	0
25	Distal femur	50	21	0	80	33	0
6	Proximal tibia	70	38	0	72	29	0
9	Proximal tibia	81	45	0	66	28	0
10	Proximal tibia	18	13	0	35	2	0
23	Proximal tibia	79	38	0	62	27	0
10	Distal tibia	48	35	0	53	8	0
22	Soft tissue at distal femur	6	9	0	2	5	0
23	Soft tissue at distal femur	0	3	6	27	5	0
25	Soft tissue at distal femur	17	0	3	34	35	0
10	Soft tissue at distal tibia	0	3	6	15	13	0
9	Abscess at calcaneus	37	17	0	22	10	0
10	Abscess plantar to phalanx IV	0	3	6	53	40	0
25	Abscess plantar to phalanx IV	24	0	1	5	8	0
26	Soft tissue plantar to phalanx V	16	15	0	22	0	25
26 *	Soft tissue plantar to phalanx V	34	17	0	18	0	23
22	Abscess at inoculation	68	55	0	30	25	0
23	Abscess at inoculation	0	3	3	14	2	0
22	Centre of abscess at inoculation	53	42	0	33	31	0
26	Lymph node	53	33	0	21	0	9
26 *	Lymph node	45	4	0	6	8	0

* Second scan of pig no. 26 (see main text)

Table S4. Volume of distribution (V_T) as determined by rev2TCM.

Pig no.	Lesion	*Uncorrected input*		*Metabolite-corrected input*	
		**Infection**	**Control**	**Infection**	**Control**
6	Distal femur	0.28	0.21	0.71	0.48
9	Distal femur	0.30	0.27	0.71	0.67
22	Distal femur	0.35	0.38	1.06	1.14
23	Distal femur	0.34	0.39	0.87	0.86
25	Distal femur	0.25	0.42	0.75	1.18
6	Proximal tibia	0.27	0.18	0.67	0.46
9	Proximal tibia	0.35	0.27	0.76	0.59
10	Proximal tibia	0.33	0.33	0.41	0.32
23	Proximal tibia	0.36	0.40	0.76	0.82
10	Distal tibia	0.28	0.21	0.35	0.26
22	Soft tissue at distal femur	0.40	0.20	2.98	0.69
23	Soft tissue at distal femur	0.59	0.35	1.63	0.78
25	Soft tissue at distal femur	0.66	0.27	4.87	1.07
10	Soft tissue at distal tibia	0.49	0.26	0.63	0.38
9	Abscess at calcaneus	0.27	0.15	0.56	0.31
10	Abscess plantar to phalanx IV	0.45	0.26	0.57	0.35
25	Abscess plantar to phalanx IV	0.53	0.41	1.77	3.02
26	Soft tissue plantar to phalanx V	0.54	0.57	2.08	2.24
26 *	Soft tissue plantar to phalanx V	0.67	0.49	3.72	4.64
22	Abscess at inoculation	0.26	0.09	0.80	0.23
23	Abscess at inoculation	0.67	0.32	1.27	1.34
22	Centre of abscess at inoculation	0.12	0.08	0.44	0.23
26	Lymph node	0.39	0.26	1.12	2.78
26 *	Lymph node	0.49	0.26	2.16	0.80

* Second scan of pig no. 26 (see main text)
